# Macrophages from naked mole-rat possess distinct immunometabolic signatures upon polarization

**DOI:** 10.3389/fimmu.2023.1172467

**Published:** 2023-04-19

**Authors:** Ekaterina A. Gorshkova, Ekaterina O. Gubernatorova, Ekaterina M. Dvorianinova, Taisiya R. Yurakova, Maria V. Marey, Olga A. Averina, Susanne Holtze, Thomas B. Hildebrandt, Alexey A. Dmitriev, Marina S. Drutskaya, Mikhail Yu. Vyssokikh, Sergei A. Nedospasov

**Affiliations:** ^1^ Engelhardt Institute of Molecular Biology, Russian Academy of Sciences, Moscow, Russia; ^2^ Belozersky Institute of Physico-Chemical Biology, Lomonosov Moscow State University, Moscow, Russia; ^3^ Center for Precision Genome Editing and Genetic Technologies for Biomedicine, Engelhardt Institute of Molecular Biology, Russian Academy of Sciences, Moscow, Russia; ^4^ Federal State Budget Institution “National Medical Research Center for Obstetrics, Gynecology and Perinatology Named after Academician V.I. Kulakov”, Ministry of Healthcare of the Russian Federation, Moscow, Russia; ^5^ Department of Reproduction Management, Leibnitz Institute for Wildlife Research, Berlin, Germany; ^6^ Division of Immunobiology and Biomedicine, Center of Genetics and Life Sciences, Sirius University of Science and Technology, Federal Territory Sirius, Krasnodar Krai, Russia

**Keywords:** naked mole-rat, macrophage, polarization, inducible NO-synthase, immunometabolism

## Abstract

The naked mole-rat (NMR) is a unique long-lived rodent which is highly resistant to age-associated disorders and cancer. The immune system of NMR possesses a distinct cellular composition with the prevalence of myeloid cells. Thus, the detailed phenotypical and functional assessment of NMR myeloid cell compartment may uncover novel mechanisms of immunoregulation and healthy aging. In this study gene expression signatures, reactive nitrogen species and cytokine production, as well as metabolic activity of classically (M1) and alternatively (M2) activated NMR bone marrow-derived macrophages (BMDM) were examined. Polarization of NMR macrophages under pro-inflammatory conditions led to expected M1 phenotype characterized by increased pro-inflammatory gene expression, cytokine production and aerobic glycolysis, but paralleled by reduced production of nitric oxide (NO). Under systemic LPS-induced inflammatory conditions NO production also was not detected in NMR blood monocytes. Altogether, our results indicate that NMR macrophages are capable of transcriptional and metabolic reprogramming under polarizing stimuli, however, NMR M1 possesses species-specific signatures as compared to murine M1, implicating distinct adaptations in NMR immune system.

## 1 Introduction

The naked mole-rat is a relatively new animal model to study immune system and immunoregulation that attracted a lot of attention due to extraordinary lifespan and apparent resistance to tumorigenesis. Recent studies including single-cell RNA-sequencing-based analysis uncovered a crucial difference in the immune organ structure and cell composition of NMR as compared to a mouse, the most frequently studied model organism in the immunological research (1–3). NMR possesses an expanded myeloid compartment comprised of granulocytes and macrophages and reduced lymphoid cell populations such as cytotoxic T-lymphocytes (CTL), B and NK cells (1, 3), in agreement with the previously reported enhanced susceptibility of these animals to viral infections (4, 5). This can be partially explained by “myeloid bias” observed in bone marrow hematopoiesis of NMR (2). Interestingly, myeloid bias in mice and humans is associated with aging of the immune system (6). However, hematopoietic stem cells of aged NMR demonstrate no age-related upregulation of inflammatory markers, remain quiescent and possess an increased cell cycle length (2), showing lack of “inflammaging” hallmarks (7), which is consistent with the concept of juvenile physiology proposed for NMR (8). In line with this, some *in vivo* studies reported that NMR is resistant to inflammation-associated diseases, such as chemically induced psoriasis (9, 10). Also, the efficiency of the innate arm of the immune system is in agreement with the ability to control gut microflora (11) by the production of highly active antimicrobial peptides (12). Based on these observations, we hypothesized that myeloid cell abundance could correlate with distinct macrophage functions and immune responses in NMR.

Macrophages are essential for maintaining both tissue defense and homeostasis. A broad spectrum of PAMP, Fc-, scavenger receptors is present on the macrophage surface and provides sensing of microenvironment. Under inflammatory conditions, macrophages secrete cytokines in order to fine-tune immune responses, and also contribute to pathogen clearance. Once the inflammation is resolved, macrophages become involved in wound healing and regeneration by absorbing damaged tissue and stimulating the repair. Tissue repair may be mediated by activation of cell proliferation with reactive oxygen species (ROS) (13). The ability to change functional phenotype under the influence of the microenvironment is known as macrophage polarization. Thus, monocyte-derived macrophages demonstrate high plasticity, while tissue-resident macrophage polarization potential may be restricted by niche-specific factors (14). Classical *in vitro* defined dichotomy of macrophages distinguishes between inflammatory (M1) and anti-inflammatory (M2). This concept is now considered as an oversimplified model (15), however, it is still useful from the evolutionary perspective (16). Characteristic traits of M1 and M2 macrophages are well-documented [reviewed in (17, 18)]. Recent studies indicate that the macrophage polarization is reciprocally regulated by the mechanisms of cell metabolism (19). Upon lipopolysaccharide (LPS) and interferon-γ (IFNγ) stimulation, M1 macrophages shift towards aerobic glycolysis and undergo so-called tricarboxylic acid cycle (TCA) breaks to meet their anabolic requirements, such as quick synthesis of antimicrobial peptides, cytokines and prostaglandins. Reactive nitrogen species (RNS) are another important component of cellular defense and regulation (20). In particular, NO participates in the suppression of mitochondrial respiration in M1 cells through direct inhibition of mitochondrial complex IV (21). At the same time deregulation of electron transport chain (ETC) drives the production of reactive oxygen species (ROS), which are indispensable for pathogen elimination. The above-mentioned inflammation-linked metabolic shift is highly conserved in metazoans (22, 23). On the other hand, anti-inflammatory functions of macrophages, such as tissue remodeling and immunosuppression, are associated with catabolic processes (oxidative phosphorylation (OXPHOS) and fatty-acid oxidation) (16). Another signature of M2-polarization is upregulation of arginase 1 which induces factors involved in tissue repair (19).

Macrophage functions are strongly associated not only with cell metabolism, but also with tissue and whole body energy requirements. Gene expression analysis suggests that NMR possesses systemic metabolic adaptations: a lower performance of the mitochondrial respiratory chain, the use of fatty acids as the main source for OXPHOS (24) and the elevated baseline levels of HIF1-α (25). Unique type of thermoregulation (26–28), resistance to anoxia, hypoxia (29), hypercapnia (30) and presence of mild depolarization of mitochondria throughout lifespan (31), are also important parameters that determine NMR as a species, whose metabolic responses to environmental stress markedly differ from most other mammals (32). However, the immunometabolic features of NMR macrophages under polarizing stimuli remain unknown. Considering the prevalence of myeloid cells in the NMR immune system and the unique metabolism, we hypothesized that macrophages may acquire some distinct adaptive features, which could be traced by the transcriptomic and immunometabolic profiles.

## 2 Materials and methods

### 2.1 Animals

Naked mole-rat bone marrow and spleen samples were collected at Lomonosov MSU (Moscow, Russia) and Leibniz Institute for Zoo and Wildlife Research (Berlin, Germany). NMR euthanasia and tissue sampling were approved by the local ethics committee of the “Landesamt für Gesundheit und Soziales”, Berlin, Germany (#ZH 156). Naked mole-rats aged 250 to 1130 days of both genders were used. C57Bl/6 mouse bone marrow, spleen, and blood samples were acquired from animals housed in SPF conditions at the Animal Facility of the Center for Precision Genome Editing and Genetic Technologies for Biomedicine, EIMB RAS (under the contract ##075-15-2019-1660 from the Ministry of Science and Higher Education of the Russian Federation). All manipulations with animals were carried out in accordance with the protocol approved by the Bioethics Committee of the EIMB RAS (Protocol No. 3 from 27/10/22).

### 2.2 LPS administration

Naked mole-rats and mice of both genders were weighed, randomly split into groups with three to five animals per group. Each group was injected *i.p.* with 50 µg LPS (Sigma-Aldrich, Germany, L2630) per 20 g body weight, whereas the control group received vehicle buffer only (PBS). 24 h after injection animals were euthanized, blood and spleens were collected for further FACS analysis.

### 2.3 CFU-C assay

Bone marrow (BM) was aseptically flushed from NMR femora, tibiae, and humeri with phosphate-buffered saline (PBS, Gibco, USA). Lysis buffer-treated BM cells (5×10^4^ cells/mL) were added to semi-liquid media with recombinant human growth factors (StemMACS HSC-CFU media with Epo, Miltenyi Biotec, Germany) and then seeded in 500 μL per well in 24-well plates. Colonies were cultured for 14 days at 32°C or 37°C and then counted. The same procedures were applied to murine BM samples.

### 2.4 Primary cultures of bone marrow-derived macrophages

Bone marrow cells aseptically flushed from NMR femora, tibiae, and humeri with phosphate-buffered saline (PBS, Gibco, USA) (0.5×10^6^ cells/mL) were cultured in high glucose DMEM (Gibco, USA) supplemented with L-glutamine, Penicillin, Streptomycin (Thermo Fisher Scientific, USA) and 10% FВS (Capricorn Scientific, Germany) in the presence of mouse recombinant M-CSF (40 ng/mL) for 7-21 days at 32°C in Petri dishes. At day 7 macrophages were detached using ice cold PBS and then seeded on 24-well plates for further activation. Macrophages (M0) were activated with a mixture of LPS (10 ng/mL; *E.coli*:O111, Sigma, USA) and recombinant mouse IFNγ (10 ng/mL; Miltenyi Biotec, Germany) for M1 polarization or recombinant mouse IL-4 (20 ng/mL; Miltenyi Biotec, Germany) for M2. In some experiments, cAMP (N6,2′-O-Dibutyryladenosine 3′,5′-cyclic monophosphate sodium salt, Sigma, USA) was used as a non-specific M2 activator. After 24 h, cells and medium were collected for RNA and protein evaluation. The same procedures were performed with 21-day NMR BMDM cultures. Mouse BMDM were cultured and activated under the same conditions except for the temperature (37°C for murine cells) in parallel with the NMR BMDM.

### 2.5 NO production

At days 7 and 21, bone marrow-derived macrophages were seeded at 0.5×10^5^ cells in 100 μl of DMEM/10% FBS in 96-well plates, and then activated using different LPS concentrations as well as standard polarization protocol. NO production was determined using Griess reagent (33) as nitrite concentration in 50 μl culture supernatant and evaluated in μM using a nitrite standard curve.

### 2.6 3-nitrotyrosine and lipid peroxidation measurement

2.5×10^5^ NMR and mouse BMDM were polarized for 24 h, and then cells were collected and frozen. Content of 3-nitrotyrosine in cell lysates was estimated with help of 3-Nitrotyrosine ELISA Kit (Abcam, USA) according to manufacturer protocol using Thermofisher “Fluoroscan” microplate reader (USA) at 600 nm. Product of lipid peroxidation 4-hydroxynonenal in lysate of macrophages from NMR and mouse was measured using to “Lipid Peroxidation Assay Kit” (Abcam, USA) and Thermofisher “Fluoroscan” microplate reader (USA) (450 nm). Calibration was done with standards provided in the kit and the values expressed as mean of three replicates with standard deviation in pmol/mg of protein.

### 2.7 RNA extraction, cDNA preparation and RT-qPCR

M0, M1, and M2 macrophages were lysed in RLT Buffer (Qiagen, Germany) followed by RNA extraction with Qiagen RNeasy Mini Kit (Qiagen, Germany) using the manufacturer’s protocol. RNA was reverse-transcribed into cDNA with RevertAid First Strand cDNA Synthesis Kit (Thermo Fisher Scientific, USA) followed by quantitative real-time PCR. qPCRmix-HS SYBR+LowROX (5X) (Evrogen, Russia) was used to amplify target genes with specific primers (Evrogen, Russia) (**Table 1**). Gene expression analysis was performed using Quant studio 6 (Applied Biosystems, USA). Relative expression level was normalized using *Actb* and calculated as 2^-ΔΔCt^ fold change to M0 (34).

**Table 1:** RT-qPCR primers used in study.

Gene	Species	Sequence (5’-3’) F	Sequence (5’-3’) R
*Actb*	*H.glaber*	GCGCTCTTTCAGCCTTCTTT	TTGGCATAGAGGTCCTTGCG
*Tnf*		ATGGCATGGATCTAACGG	CGGCTGACAGTATGGGTG
*Fpr2*		CAGATCACCAAGCCATTGCC	GCAACAAGAAGGGGCCGTAG
*Cd38*		AATTACAGTGACTCATGCTCAG	TCCAACACAAATGTGACTCAG
*Arg1*		CATCGGAGCCCCTTTCTCAA	ACCAGCATATCTCAACGCCG
*Egr2*		AATCTGCCCCCTTCTTTCGG	CCACTCCGTTCATCTGGTCA
*Mrc1*		AGCTTTGACTGCCTCGACTG	GTGGTCTTGTGTATTCACCTTTTGT
*Actb*	*M.musculus*	CTCCTGAGCGCAAGTACTCTGTG	TAAAACGCAGCTCAGTAACAGTCC
*Arg1*		TGAGGAAAGCTGGTCTGCTG	GGCCAGAGATGCTTCCAACT

### 2.8 Multiplex immunoassay

The supernatants from 2.5×10^5^ control or activated BMDM were analyzed with MILLIPLEX MAP Mouse Cytokine/Chemokine Magnetic Bead Panel-Premixed 32 Plex Kit (Merck, Germany) according to the manufacturer’s protocol.

### 2.9 Combined metabolic stress test

Extracellular flux analysis of polarized BMDM was performed by measuring oxygen consumption rate (OCR) and extracellular acidification rate (ECAR) using a Seahorse XFe24 extracellular flux analyzer (Agilent, USA). Based on the standard manufacturer’s protocol, we optimized cell density and carbonyl cyanide-4-trifluoromethoxy-phenylhydrazone (FCCP) concentrations to measure OCR and ECAR in NMR macrophages and adjusted all incubation steps in the protocol to 32°C. To measure OCR and ECAR, culture medium of 2.5×10^5^ macrophages/well-polarized for 24 h in XF24 Cell Culture Microplates (Agilent, USA) was replaced with 500 μl non-buffered minimal DMEM (Agilent, USA) supplemented with 2 mM L-glutamine (Gibco, USA) and 2 mM pyruvate (Sigma, USA) at pH 7.4. After incubation without CO_2_ at 32°C for 45 min, OCR and ECAR were measured at basal level and under consequent adding of glucose (10 mM), oligomycin (1 μM), FCCP (2 μM) and mixture of Rotenone (1 μM), Antimycin A (1 μM) and 2-deoxy-glucose (5 mM) (Sigma, USA). Three measurement cycles following each injection consisted of 3 min mixing, 1 min waiting, and 2 min measuring. A minimum of three technical repeats were used for each polarization condition.

### 2.10 Flow cytometry

NMR and mouse splenocytes, blood, BM, and BMDM were stained with a Fixable Viability Dye and the panel of antibodies suitable for NMR tissues (**Table 2**). Rat anti-mouse CD16/32 Ab (clone 93) was used for blocking of unspecific binding. Washing steps were performed with PBS/2% FВS. The data were acquired using a BD FACS Canto II flow cytometer. For intracellular nitric oxide staining DAF-FM diacetate (dilution factor 2000) (Thermo Fisher Scientific, USA) was added to Abs mix.

**Table 2:** Fluorophore-conjugated antibodies to mouse and NMR immune cell markers used in the cytometric assay.

Marker	Fluorophore	Clone		Reference
		Mouse	NMR	
CD11b	PerCP-Cy5.5	M1/70 (Thermo Fisher)		(35, 36)
CD14	APC	Sa14-2 (Thermo Fisher)	TM1 (DRFZ in house)	(37)
CD8	FITC	53-6.7 (Thermo Fisher)	CT6 (Bio-Rad)	(37)
CX3CR1	bv421	SA011F11 (Biolegend)		Previously not described

### 2.11 Mitochondria staining

BMDM were seeded on confocal microscopy sectored dishes (5×10^4^ cells per sector) and then M1/M2-polarized as described above. Cells were washed following stimulation and stained with 100 nM MitoTracker Green FM, and 2 μg/mL Hoechst 33342 (Sigma Aldrich, USA) for 30 min. Microscopy was performed using Leica TCS SP5 (Leica, Germany) confocal microscope. For analysis of mitochondrial network morphology, Image-J compatible MiNA tool was used (38).

### 2.12 Statistical analysis

Cellular composition of immune organs, surface phenotypes of BMDM cultured under different temperatures, and gene expression and cytokine production of polarized BMDM were analyzed in groups of 3-6 animals at least in two independent experiments. CFU-C and metabolic stress test experiments were performed at least three times with individual bone marrow-derived cultures. Data were analyzed using the GraphPad Prism 8 software. Data were first tested for Gaussian distribution with the D’Agostino & Pearson omnibus normality test and then analyzed using Student’s unpaired t-test, paired t-test, one-way ANOVA test, RM-ANOVA or two-way RM-ANOVA tests, or mixed-effect analysis followed by Sidak’s or Tukey’s post-test analysis for multiple comparisons. Results are displayed as mean ± SD. Differences were considered significant when p-values were below 0.05.

### 2.13 RNA-sequencing

Twelve RNA samples of four differently activated macrophage cultures were prepared using Qiagen RNeasy Mini Kit with DNAse I (Qiagen, Germany) following the manufacturer’s protocol. RNA concentrations were measured using Qubit RNA BR Assay Kit and Qubit 4 fluorometer (Thermo Fisher Scientific, USA). RNA quality control was performed using Agilent Bioanalyzer 2100 and Agilent RNA 6000 Nano kit (Agilent, USA). For the cDNA library preparation, RNA samples (300 ng per sample) were first enriched with mRNA by NEBNext Poly(A) Magnetic Isolation Module (NEB, USA) and then processed using NEBNext Ultra II Directional RNA Library Prep Kit for Illumina (NEB, USA). For sample multiplexing, NEBNext Multiplex Oligos for Illumina (Index Primers Set 1) (NEB, USA) were used. cDNA library size of approximately 300 bp was verified by Agilent Bioanalyzer 2100 and Agilent DNA 1000 kit (Agilent, USA). RNA sequencing was performed on Illumina NextSeq 550 via NextSeq 500/550 High Output Kit v2.5 (75 Cycles) (Illumina, USA). The obtained data were deposited in the NCBI Sequence Read Archive (SRA) under the BioProject accession number PRJNA933639.

### 2.14 Differential expression analysis

Transcriptomic analysis was conducted using the PPline (39) software and included the following steps. Transcriptomic reads were examined for rRNA presence (*H. glaber* rRNA sequences were downloaded from https://rnacentral.org/search?q=Heterocephalus%20glaber%20rRNA) and bacterial contamination (bacterial genomes assembled before 2015 were downloaded from the NCBI database). The alignment of the sequencing reads to the compiled databases was performed with bowtie2 (40). Trimmomatic-0.38 was used to trim off the adapter sequences and trim transcriptomic reads by quality (trailing:24, slidingwindow:4:14, minlen:40) (41). The alignment of the *H. glaber* GCA_000247695.1 genome (https://hgdownload.soe.ucsc.edu/hubs/GCA/014/060/925/) was performed with STAR (42). FeatureCounts from the subread 1.6.0 package (43) and the *H. glaber* annotation file (GCF_000247695.1) were used to calculate feature counts per *H. glaber* gene. Further analysis of differentially expressed genes and Venn diagram plotting were performed with RTrans (https://github.com/gskrasnov/RTrans), which is based on the edgeR 3.28.1 package (44). The TMM-normalization method and QLF-tests were used. A differentially expressed gene was included in a Venn diagram if it had statistics below the chosen thresholds: strict p-value = 0.01 (QLF-test), mild p-value = 0.05 (QLF-test), strict absolute logFC value = 0.5, mild absolute logFC value = 0.3, strict logCPM = 1.5, strict logCPM = 1.0. To be included in the intersection, the statistics had to be superior to the strict thresholds in at least one comparison and superior to the mild thresholds in another. For differentially expressed genes with p-value < 0.01 (QLF-test), dot plots were manually created in R 4.2.2 using the ggplot package. For downregulated and upregulated genes displayed in the Venn diagrams, GO analysis was conducted with the DAVID online-tool (https://david.ncifcrf.gov/). For *M. musculus*, DE of the publicly available data was analyzed similarly to NMR. The GRCm38 (Ensembl release 102) version of the *M. musculus* genome and the GRCm38.p6 annotation were used. Sequencing runs were downloaded from SRA (PRJNA407775: SRR6048662, SRR6048663, SRR6048664, SRR6048674, SRR6048675, RR6048676; PRJNA669983: SRR12844286, SRR12844287, SRR12844288, SRR12844289, SRR12844290, SRR12844291).

## 3 Results

### 3.1 Distinct temperature requirements for NMR macrophage cultures *in vitro*

**Figure 1:**
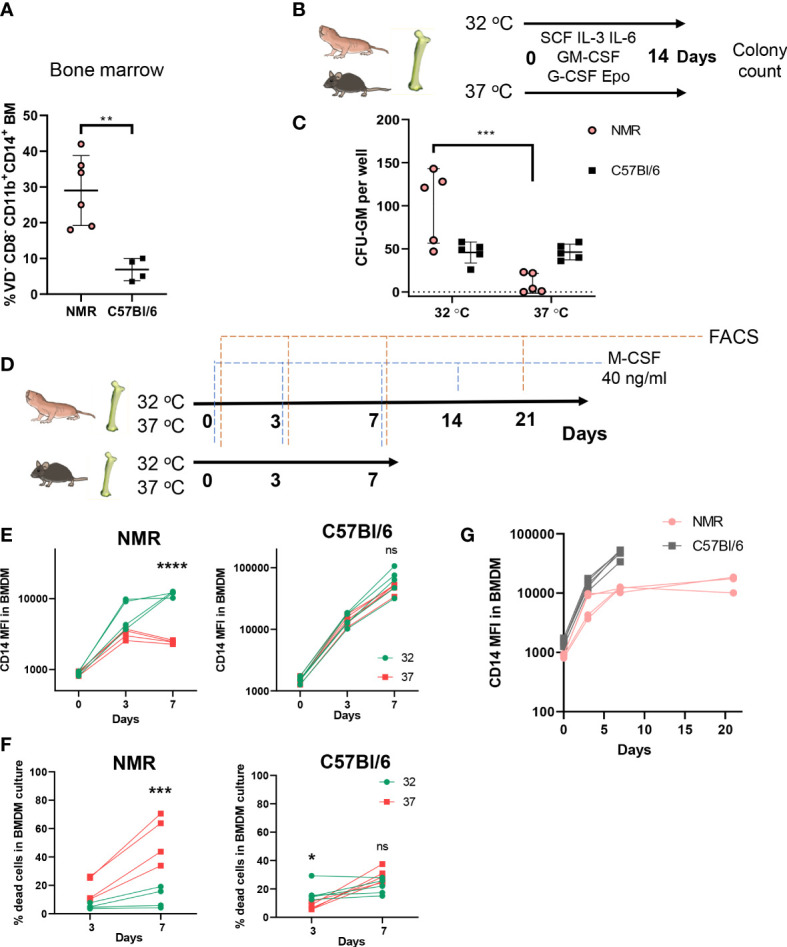
Cellular composition of hematopoietic organs and susceptibility of myeloid progenitors to temperature suggests unique adaptations of NMR myeloid cells. **(A)** Percent of myeloid CD11b^+^CD14^+^ cells in bone marrow of NMR and C57Bl/6 mouse; **(B)** The ability of NMR BM progenitors to differentiate at two temperatures was determined by CFU-C assay: non-lysed BM cells were seeded in semiliquid medium with growth factor mixture and cultured for 14 days at 32°C and 37°C, CFUs were identified by morphology and counted at the day 14; **(C)** CFU-GM count at day 14 and 32°C or 37°C in NMR and C57Bl/6 mouse bone marrow cultures; **(D)** Cells, isolated from NMR and mouse BM, were seeded in recombinant mouse M-CSF-containing DMEM/10%FBS medium and cultured for 7 days at 32 and 37°C. In two experiments NMR cells were cultured at 32°C for 21 days. Fresh medium was added to cells at days 3, 7, 14, 18. FACS analysis of BMDM cultures was performed at days 0, 3, 7, 21. **(E)** CD14 MFI on live CD11b^+^ cells at days 0, 3, 7 during NMR or mouse BMDM maturation at 32°C (green) or 37°C (red). **(F)** Percent of dead cells stained with Viability Dye at days 0, 3, 7 during NMR or mouse BMDM maturation at 32°C (green) or 37°C (red). **(G)** CD14 MFI on CD11b^+^ cells during BMDM maturation at day 0, 3, 7, 21 for NMR and day 0, 3, 7 for murine cells. Results displayed as mean ± SD.**p<0.01 Student’s unpaired t-test; *p < 0.05; ***p < 0.001; ****p < 0.0001; ns, not significant, two-way RM ANOVA, Šidak’s multiple comparisons test.

Defining the cellular composition and the abundance of various immune cell populations in the NMR remains difficult due to a limited number of specific antibodies applicable for flow cytometry and immunohistochemistry. Previously, several such cross-specific antibodies for NMR myeloid cells were described (35–37). Using this panel, we established that NMR bone marrow is characterized by a higher proportion of CD11b^+^ CD14^+^ cells as compared to mice (**Figure 1A**). We also analyzed density gradient separated bone marrow fractions to determine, which subpopulations contribute to the increase in the proportion of CD14^+^. We concluded that the majority of CD11b^+^ CD14^+^ cells were comprised of myeloid progenitor cells and monocytes, and not mature neutrophils (**Supplementary Figure 1A**). We hypothesized that the abundance of CD14^+^ cells may reflect the bias of NMR myeloid precursors to antibacterial responses. To further address this idea *in vitro* we established bone marrow-derived macrophage (BMDM) cultures.

First, we compared and optimized published protocols for NMR BMDM culture conditions (35, 45). To define optimal temperature for BMDM differentiation we assessed the number of myeloid colony-forming units (CFU) in semi-liquid methylcellulose media supplemented with human recombinant growth factors (SCF, GM-CSF, G-CSF, IL-3, IL-6, Epo) at 32°C (physiologically relevant NMR body temperature) or 37°C (standard temperature for *in vitro* cell culture experiments) at 5% CO_2_. Naked mole-rat and mouse bone marrow cells were cultured for 14 days in parallel at these two temperatures (**Figure 1B**). The largest numbers of naked mole-rat CFU-GM were formed at 32°C, whereas no colony formation was observed at 37°C in NMR bone marrow cultures (**Figure 1C**). At the same time, the numbers of myeloid colonies in mouse bone marrow cultures were not dependent on temperature conditions (**Figure 1C**). This observation led us to the hypothesis that higher temperatures suppressed differentiation or decreased the viability of NMR myeloid progenitors. To further address this, we cultured NMR and mouse BM cells in the presence of recombinant M-CSF at 32°C and 37°C followed by FACS analysis at days 0, 3 and 7 (**Figure 1D**). Surface expression of CD14 increased over this time period in NMR-derived bone marrow cultures similarly to mouse BMDM, with the peak in the mean fluorescence intensity (MFI) observed at day 7, but only at 32°C and not at 37°C culture conditions (**Figure 1E**). Also, the percentage of dying cells was significantly increased when NMR BMDM were cultured at 37°C rather than at 32°C (**Figure 1F**). Thus, we concluded that higher temperatures interfere with normal differentiation and/or survival of NMR bone marrow cells *in vitro*. All further experiments were performed under physiologically relevant temperatures: 32°C for NMR and 37°C for mouse BMDM.

Considering that NMR macrophage differentiation appeared more efficient at lower temperatures, we hypothesized that this may be accompanied by reduced cell proliferation and/or delayed differentiation. Thus, we tested whether NMR BMDM can be cultured for a longer time period. Visual assessment of NMR BMDM, cell counts and flow cytometric analysis of cells cultured with the M-CSF at 32°C for 7, 14 and 21 days were performed (**Figure 1D**). At day 7 we detected adherent cells with macrophage-like morphology (**Supplementary Figure 1C**), which expressed both CD11b and CD14 (**Figure 1E**), however, at this timepoint a large number of bone marrow cells remained in suspension. By day 21 almost all cells in NMR BMDM culture became attached. At the same time, MFI of CD14 expression in NMR BMDM at day 21 was only slightly higher than at day 7 (**Figure 1G**). We concluded that macrophages harvested at day 7 may represent a more differentiated pool of myeloid progenitor cells, whereas macrophages harvested at day 21 were derived from earlier myeloid precursors. Therefore, in most of the following experiments we tested both populations. Since NMR macrophages showed distinct differentiation dynamics as compared to mouse cells, we next addressed the potential NMR BMDM to be polarized in response to M1 and M2 stimuli.

### 3.2 NMR macrophages demonstrate less pronounced M1 polarization

**Figure 2:**
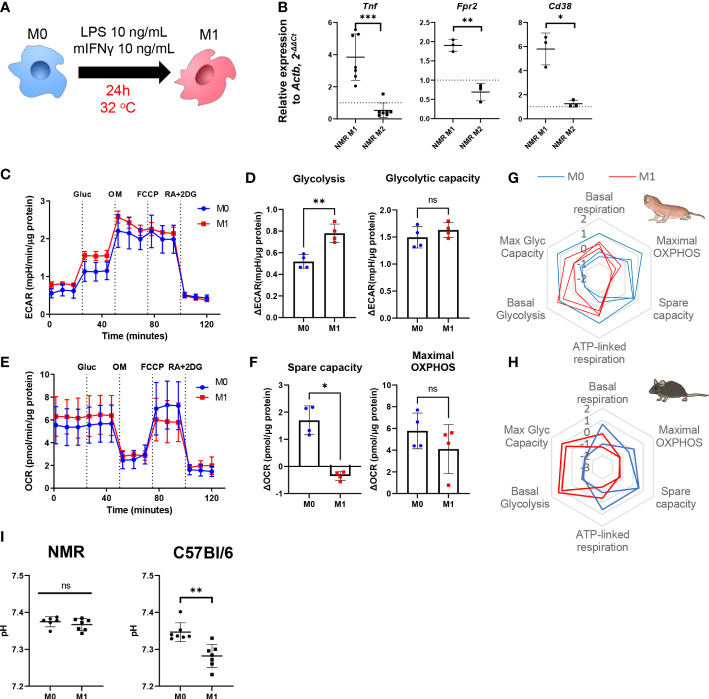
NMR macrophages demonstrate less pronounced M1 polarization. **(A)** To induce M1 polarization NMR BMDM were activated in the presence of LPS (10 ng/mL) and mouse recombinant IFNγ (10 ng/mL) for 24 h, then cells were subjected to metabolic stress test or collected for RNA extraction, control mouse BMDM were tested in parallel in the same conditions except for temperature (37°C); **(B)** Relative expression of M1-associated genes (*Tnf*, *Fpr2*, *Cd38*) in M1 and M2-conditioned NMR BMDM as a fold change to the expression level observed in M0; **(C)** Representative diagram of the real-time changes in extracellular acidification rate (ECAR) of M0 and M1 NMR macrophages and parameters of glycolysis calculated on the basis of it **(D)**; **(E)** Representative diagram of the real-time changes in oxygen consumption rate (OCR) of M0 (blue) and M1 (red) NMR macrophages upon consequently added glucose (Glu), oligomycin (OM), FCCP and mixture of Rotenone, Antimycin A and 2-deoxy-glucose (RA+2DG) and parameters of cellular respiration calculated on the basis of it **(F)**. Summary of metabolic profiles of NMR **(G)** and murine **(H)** M0 (blue) and M1 (red) macrophages represented as z-score transformed mean for six metabolic parameters on radar chart, each line on a diagram represents an independent metabolic stress experiment. **(I)** pH level in NMR and mouse M1 supernatants measured after 1 h in media supplemented with glucose using Agilent Seahorse XFe24 Analyzer. Results displayed as mean ± SD.*p<0.01; **p<0.01; ***p<0.001; ns, not significant, Student’s paired t-test or Mann-Whitney test.

To generate proinflammatory macrophages (M1) we activated NMR BMDM harvested at days 7 or 21 (M0) using a mixture of LPS and recombinant IFNγ for 24 h and then analyzed their gene expression and metabolic profiles (**Figure 2A**). In parallel mouse BMDM were used as reference of well-defined canonical polarization. We detected an unambiguous expression pattern for M1-associated genes: *Tnf, Fpr2, Cd38* were upregulated in M1 versus M0 samples (**Figure 2B**).

**Figure 3:**
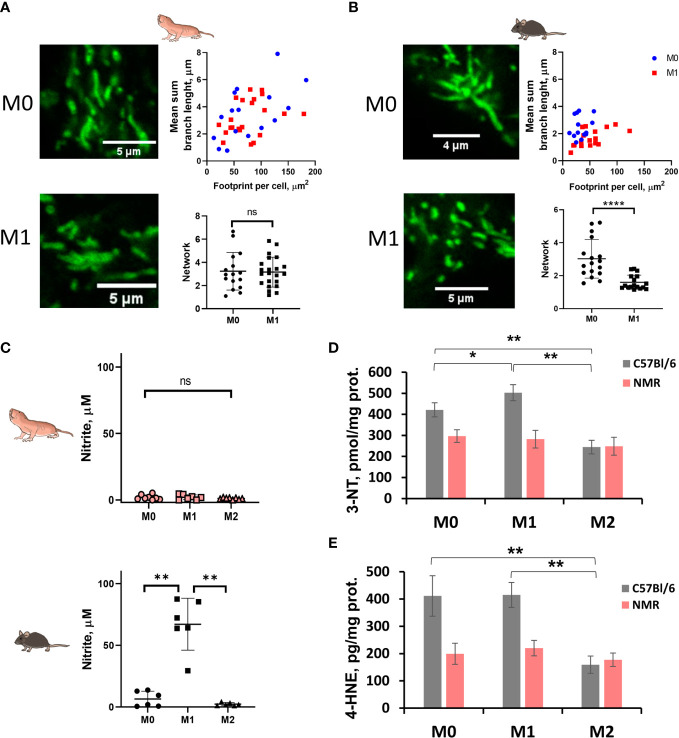
M1-polarized NMR macrophages are characterized by significant reduction in NO production accompanied by less pronounced mitochondrion fission. Representative image of MitoTracker Green stained mitochondria of M0 and M1 NMR **(A)** and murine macrophages **(B)** and their structural parameters analyzed with MiNA software for ≥20 individual cells. **(C)** Nitrite concentration in supernatants of NMR BMDMs activated by LPS (10 ng/mL) and mouse recombinant IFNγ (10 ng/mL) (M1) or mouse recombinant IL-4 (M2) for 24 h. Nitrite concentration in control mouse BMDMs activated in the same conditions except for temperature (37°C); **(D)** 3-nitrotyrosine content in cell lysates of NMR and mouse BMDMs activated by LPS (10 ng/mL) and mouse recombinant IFNγ (10 ng/mL) (M1) or mouse recombinant IL-4 (M2) for 24 h **(E)** 4-hydroxynonenal concentration in cell lysates of NMR and mouse BMDMs. Results displayed as mean ± SD. *p<0.05; **p< 0.01; ****p<0.0001; ns-not significant, one-way RM-ANOVA or Student’s t-test.

As expected, in the combined metabolic stress test NMR M1 cells increased the rate of glycolysis following glucose injection as compared to M0 (**Figures 2C, D, G**; **Supplementary Figures 2A, B**). Maximal respiration and spare capacity of mitochondrial respiration were decreased in NMR M1 cells (**Figures 2E, H**) in similar manner as in murine M1 cells (**Figure 2H**). However, metabolic profiles of M1 and M0 macrophages demonstrated that NMR BMDM underwent a less dramatic glycolytic shift and showed reduced mitochondrial disfunction compared to mouse cells upon inflammatory activation (**Figure 2G**, **Supplementary Figures 2A, B**). Supernatants from NMR M1 cells retained the same pH as M0 cells, unlike murine M1 cells, which lowered pH due lactate accumulation (**Figure 2I**). Finally, we analyzed mitochondrial morphology of M1 macrophages using MitoTracker Green. To define complexity of mitochondrial structures, we evaluated mitochondrial footprints, numbers and mean lengths of branches in networks (37). Mitochondrion of NMR M1 cells showed a very similar morphology to M0 cells (**Figure 3A**), with no obvious signs of fission with network pruning and branch shortening as reported for murine M1 cells (**Figure 3B**).

Taken together, gene expression analysis confirmed the canonical M1 polarization profile in response to LPS and IFNγ, while extracellular flux experiments and mitochondrial staining both indicated that mitochondrion in NMR M1 cells were functional, suggesting a less pronounced M1 immunometabolic phenotype.

### 3.3 M1-polarized NMR macrophages are characterized by significant reduction in NO production

Production of free radicals by M1 macrophages is one of important antimicrobial defense mechanisms. Nevertheless, excessive amounts of reactive oxygen (ROS) or nitrogen species (RNS) can cause damage to the host tissues, so their production is tightly regulated. The level of nitrites in supernatants from activated cells were analyzed. Surprisingly, we found that NMR M1 macrophages produced low or even undetectable amounts of NO after 24 h of activation, unlike mouse M1 macrophages (**Figures 3C, D**; **Supplementary Figure 2C**). To prove that the absence of nitrites in NMR samples was not dependent on a particular activation protocol, we tested supernatants collected at 2, 8, 24 and 48 h after activation with LPS (100 ng/mL) or LPS (100 ng/mL)/IFNγ (50 ng/mL). Remarkably, as in the case of standard M1 activation, we did not detect a significant increase of nitrites in any of NMR samples (**Supplementary Figure 3A**). On the other hand, NO levels in mouse samples increased dramatically over time and were dependent on the concentrations of LPS and the presence of IFNγ (**Supplementary Figure 3A**). Additionally, we stained M0 and M1 NMR macrophages using DAF-FM diacetate, but failed to detect any increase in DAF-FM MFI in NMR samples (**Supplementary Figure 3B**).

It is known that NO can quickly react with superoxide anion radical at diffusion-controlled rates (~1 × 10^10^ M^–1^ s^–1^) to form peroxynitrite (PN) unstable at neutral pH in the cell (NO• + O_2_• → ONOO^–^) (46). NO• is a stable but highly diffusible free radical, thus, location of PN formation is thought to be associated with the superoxide production sites. Since the decomposition products of peroxynitrite are highly reactive radicals, potential targets of peroxynitrite include lipids as well as proteins. Thus, we measured the stable modification product of oxidative damage to tyrosine (3-nitrotyrosine, 3-NT) to indirectly judge the initial level of peroxynitrite and NO• in NMR and mouse BMDM. Interestingly, while for mouse M1 BMDM an increase in 3-NT was observed (**Figure 3D**, grey columns), 3-nitrotyrosine levels in mouse M2 BMDM were decreased. NMR macrophages demonstrated no significant differences in the 3-NT content between M0, M1 and M2 (**Figure 3D**, pink columns), which correlated with the lack of changes in NO levels in NMR BMDM culture media (**Figure 3C**). To evaluate whether similar 3-NT levels in differently activated NMR BMDM were associated with higher production of superoxide in these cells, we measured the level of the lipid peroxidation product 4-hydroxynonenal (4-HNE) in cell lysates. 4-HNE level was lower for mouse M2 macrophages compared to M0 and M1 (**Figure 3E**). At the same time, no significant differences in 4-HNE were observed for any type of NMR macrophages, which corresponds to the observation of a similar level of 3-nitrotyrosine, reflecting oxidative damage to proteins during the formation of secondary nitrosyl radicals (**Figure 3E**).

**Figure 4:**
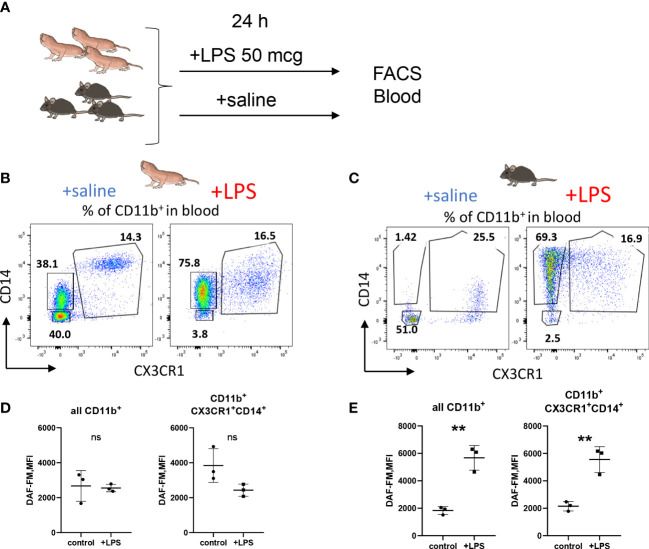
NO production is reduced in NMR myeloid cells after LPS administration *in vivo.* **(A)** Groups of animals (n≥3) were subjected to LPS administration *i.p.* (50 mcg/20 g bodyweight), after 24 h peripheral blood was analyzed by FACS. Representative dot plots of NMR **(B)** and mouse **(C)** CD11b^+^ blood cells distributed via CD14 and CX3CR1 surface expression in control and LPS groups. DAF-FM MFI measured in whole CD11b^+^ blood population and in the population of CX3CR1-positive monocytes in NMR **(D)** and mouse **(E)**. Results displayed as mean MFI ± SD. **p<0.001; ns-not significant, Student’s t-test.

To further support the finding of limited RNS production by NMR macrophages we also evaluated NO production by NMR blood myeloid cells 24h after LPS administration *in vivo* (**Figure 4A**). There was no DAF-FM MFI increase in the entire CD11b^+^ population or in CD11b^+^ CD14^+^ CX3CR1^+^ subpopulation, presumably, comprising blood monocytes (**Figures 4B, C**; **Supplementary Figure 3C**) in agreement with *in vitro* data (**Figure 3**). On the other hand, mouse blood monocytes and granulocytes demonstrated an increase in DAF-FM MFI as expected (**Figures 4D, E**). Altogether, we observed limited ability of NMR inflammatory macrophages to produce RNS.

### 3.4 Impaired M2 polarization of NMR macrophages in response to IL-4

**Figure 5:**
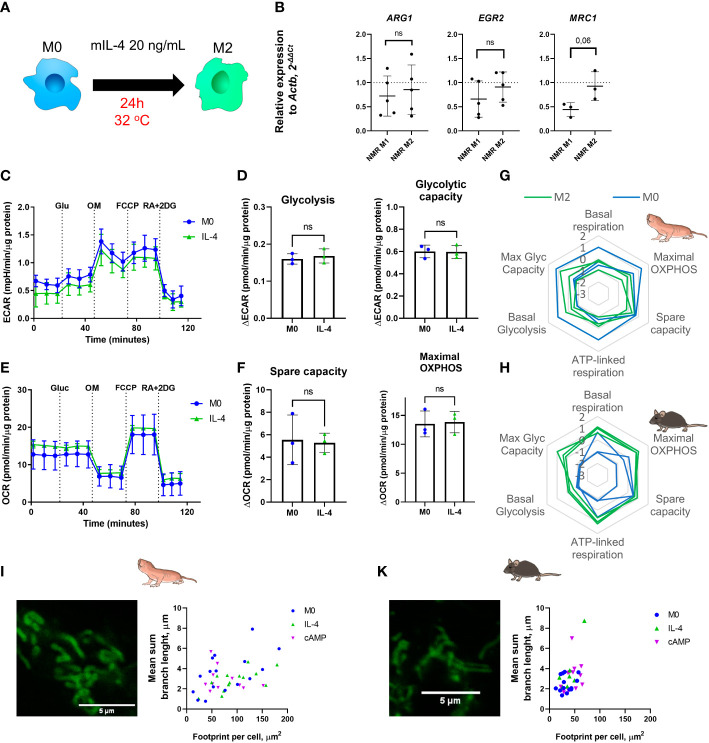
Recombinant mouse IL-4 triggers non-canonical M2 polarization profile in naked mole-rat BMDM cultures. **(A)** To induce M2 polarization NMR BMDM were incubated with mouse recombinant IL-4 (20 ng/mL) for 24h, then cells were subjected to metabolic stress test or collected for RNA extraction, control mouse BMDM were tested in parallel; **(B)** Relative expression of M2-associated genes in M1 and M2-conditioned NMR BMDM as a fold change to the expression level observed in M0; **(C)** Representative diagram of the real-time changes in extracellular acidification (ECAR) of M0 and M1 NMR macrophages and cellular glycolysis parameters **(D)**. Representative diagram of the real-time changes in OCR of M0 (blue) and M2 (green) NMR macrophages under combined stress test **(E)** and parameters of cellular respiration parameters **(F)**; **(G)** Summary of metabolic profiles of NMR and murine **(H)** M0 (blue) and M2 (green) macrophages represented as z-score transformed mean for six metabolic parameters on radar chart, each line on a diagram represents an independent metabolic stress experiment. **(I)** Representative image of MitoTracker Green stained mitochondria of NMR and murine **(J)** M2 macrophages and their structural parameters. Results displayed as mean ± SD; ns-not significant, Student’s paired t-test or Mann-Whitney test.

To generate alternatively activated or anti-inflammatory macrophages (M2) recombinant mouse IL-4 for 24 h to NMR BMDM harvested at the day 7 or 21 (M0) was added. Gene expression, cytokine production and metabolic profiles were then analyzed (**Figure 5A**). The expression of M2-associated genes (*Arg1*, *Egr2*, *Mrc1*) was not changed in M2 compared to M0 NMR macrophages (**Figure 5B**). As mentioned above, alternatively activated macrophages retain the TCA cycle intact and rely on OXPHOS as the main metabolic pathway. We found that glycolytic capacity of mouse M2 was also elevated over M0 in combined metabolic stress tests (**Figure 5H**). However, in IL-4-stimulated NMR macrophages we observed unexpected metabolic profiles (**Figure 5G**) characterized by the absence of increase in maximal OXPHOS and spare capacity, while undergoing polarization from M0 to M2 (**Figures 5E, F, G**; **Supplementary Figures 2A, B**). Unlike in the mouse system, maximal glycolytic capacity was not elevated in M2 as compared to M0 (**Figures 5C, D, G**). Overall, the metabolic phenotype of NMR M2 cells appeared more quiescent, rather than energetic, as suggested by the results of extracellular flux experiments (**Figures 5G, H**).

To evaluate whether mitochondria from NMR M2 macrophages have peculiar morphology as compared to mice, we examined the mitochondrion structure. Both IL-4- and cAMP-stimulated M2 NMR macrophages harbored mitochondria with features not significantly different from M0 cells (**Figures 5I**, **3A**), while mouse M2 cells possessed longer branches in their mitochondrion structures (**Figure 3B**, **5J**), in agreement with increased respiration parameters (**Figure 5H**). Taken together, NMR M2 macrophages were distinct from alternatively activated mouse macrophages, displaying no signs of mitochondrial fusion.

**Figure 6:**
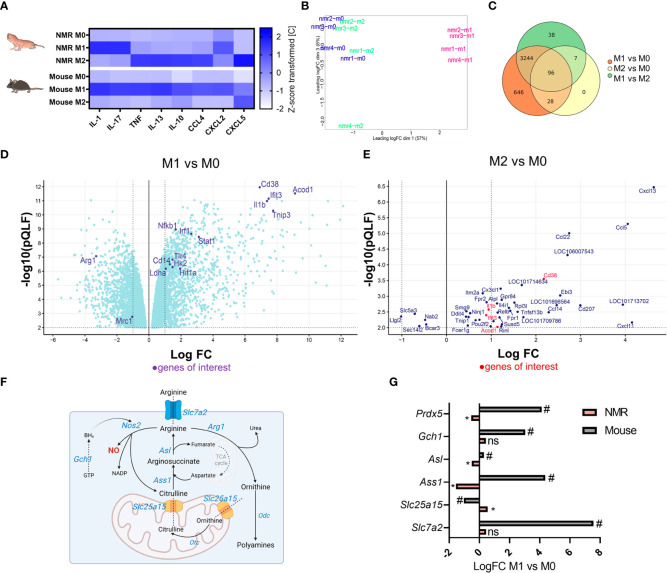
Distinct transcriptional and secretion profiles of NMR M1 and M2 macrophages **(A)** Multiplex immunoassay on the samples of M0, M1, and M2 supernatants of NMR (n=5) and mouse (n=3) macrophages, each cell shows mean of z-score transformed cytokine concentration in medium (pg/mL). **(B)** MDS plot for NMR M0, M1, M2 transcriptomic profiles; **(C)** Venn diagrams of DEGs in NMR M0, M1, M2 **(D)** Volcano plot of DEG in NMR M1 to M0, only DEG with p < 0.01 (QLF test) are displayed **(E)** Volcano plot of DEG in NMR M2 to M0, only DEG with p < 0.01 (QLF-test) are displayed. **(F)** NO-Urea cycle and aspartate-argininosuccinate shunt schematic representation with the depiction of genes involved and their logFC in M1 as compared to M0 cells in NMR (pink) and mouse (grey) **(G)**. * p<0.05 (QLF-test) in NMR; # p<0.05 (QLF-test) in mouse, ns, not significant.

In order to evaluate cytokine expression profiles of NMR and mouse polarized macrophages 32-plex immunoassay was performed. Ten analytes (**Supplementary Figure 5**) demonstrated a reliable cross-specific signal in NMR samples. Interestingly, NMR M2, but not M1, cells produced conventional M1-associated cytokines, such as IL-1β, TNF, CCL4, CCL5 (**Figure 6A**; **Supplementary Figures 2C**; **5**). In turn, NMR M1 macrophages were less active in cytokine secretion. There was a significant increase in IL-17 and IL-12p40 production, while IL-1β, TNF and CXCL2 were only slightly elevated in M1 supernatants (**Supplementary Figure 5**). Thus, we concluded that despite the absence of *Arg1* upregulation and canonical metabolic signature, NMR M2 macrophages indeed responded to recombinant IL-4. However, we cannot exclude that the activation of IL-4R was not fully efficient, as compared to the mouse system.

### 3.5 NMR M1 macrophages show species-specific changes in the expression of inflammation-associated arginine metabolism genes

Metabolic and cytokine secretion profiles of NMR BMDM stimulated with standard M1 and M2 activators prompted us to address the immune-associated functions of NMR M1 and M2 macrophages at transcriptome level. To this end, RNA-sequencing of M0, M1 and M2 cells from 21-day differentiated NMR BMDM cultures were performed. Publicly available relevant RNA-seq datasets for polarized mouse macrophages were used for comparison (GSE103958 (47); GSE159628 (48)). Multidimensional scaling of NMR group datasets uncovered a small difference between M0 and M2, while M1 was clearly distanced from other groups (**Figure 6B**). Upregulated and downregulated genes in M1 vs M0 samples represented LPS-dependent (*Nfkb1*, *Cd14*, *Tlr4*, *Lyn*, *Tnip3*, *Pik3ap1* upregulation) and IFNγ-dependent signatures (*Irf1*, *Stat1*, *Cd38*, *Ifit3* upregulation) (**Figure 6D**; **Supplementary Tables 1**, **5**). This analysis confirmed that NMR macrophages were responsive to LPS/IFNγ and that the lack of NO-synthase induction, known to be NF-κB-dependent in canonical M1 macrophages, is a distinct feature of M1 NMR macrophages (**Figure 3**). To further support this finding, RNA-seq datasets of NMR M1 cells included just a few reads aligned with NMR *Nos2* sequence. In addition, *Gch1* and *Slc7a2* expression was not elevated in NMR M1 BMDM, unlike in the mouse system (**Figure 6G**, **Supplementary Table 3**). On the other hand, one of the top upregulated genes in NMR M1 cells was *Acod1* whose product is associated with itaconate production and the so-called “first TCA cycle break” (**Figure 6D**; **Supplementary Table 1**). Together with the increased expression of *Hif1a*, *Hk2*, *Ldha* (**Figure 6D**) this increases correlated with glycolysis induction in NMR M1 cells (**Supplementary Figure 2A**).

At the same time, IL-4-activated cells had almost the same transcription profiles as control samples (**Figure 6B**) and no uniquely altered genes (**Figure 6C**, **Supplementary Table 2**). In agreement with gene expression analysis (**Figure 5B**), RNA-seq failed to show any differences in *Arg1*, *Egr2*, *Mrc1* expression levels between M2 and M0 NMR cells (**Figure 6E**, **Supplementary Table 2**), while these genes were significantly downregulated in M1 macrophages (**Figure 6D**). Overall, the observed DEGs did not resemble the canonical M2-associated signature in the mouse (**Supplementary Table 4**). IL-4-activated NMR cells demonstrated only few commonly upregulated or downregulated genes with M1 macrophages. Most of them were associated with chemotaxis (*Cxcl13*, *Ccl5*, *Ccl22*) (**Figure 6E**, **Supplementary Table 5**), consistent with the results of multiparametric immunoassay (**Figure 6A**, **Supplementary Figure 2C**).

Altogether, analysis of M1 transcriptome showed that despite the apparently intact LPS/IFNγ-response pathways, NMR inflammation-associated arginine metabolism was altered, specifically, via low level of NO-synthase expression in M1 macrophages (**Figures 3**, **6**). RNA-seq data of M2 cells did not allow us to conclude whether responsiveness of NMR macrophages to recombinant IL-4 was sufficient for transcriptome modulation.

## 4 Discussion

The present study focused on phenotypical and functional characterization of primary NMR macrophages under polarizing stimuli in comparison with pro- and anti-inflammatory populations of mouse bone marrow-derived macrophages.

One of our initial observations was based on a panel of FACS-compatible antibodies. This panel included previously described cross-specific antibodies, which recognize different epitopes on NMR immune cells (35–37), as well as newly identified cross-specific anti-mouse CX3CR1 (clone SA011F11), reported in this study. The higher abundancy of CD14^+^ myeloid cells in the bone marrow was noted in NMR as compared to laboratory mice (**Figure 1A**). Recently, NMR bone marrow composition and hematopoietic landscape were thoroughly characterized (2). According to scRNA-sequencing, the most enlarged population of NMR bone marrow cells is represented by granulocytes and granulocyte progenitors. However, in our experiments CD11b^+^ CD14^+^ population was enriched by myeloid progenitors and monocytes with fewer mature granulocytes (**Supplementary Figures 1A, B**). CD14 is a co-receptor for TLR4, which is involved in LPS/receptor complex recognition and internalization, and it also regulates LPS response (49). According to a previous study (50), CD14 baseline levels were significantly higher in neonate human monocytes as compared with those of adults. We also detected an increase in CD14^+^ population in the bone marrow of P2-P3 mice (**Supplementary Figure 1C**). Thus, we hypothesize that high abundance of CD14^+^ myeloid cells in NMR BM may refer to the general neonate state (8). Other studies did not report the difference between juvenile and adult TLR4/CD14 surface densities, but revealed lower levels of cytokine production in LPS-stimulated antigen-presenting cells in newborns as compared to adults (51). The anti-pathogen branch of neonate immune system appeared underdeveloped making animals susceptible to infections, but, at the same time, flexible enough to provide peripheral tolerance (51). In line with this, NMR is surprisingly susceptible to certain viruses (4, 5), protozoans (52) and skin bacteria, which cause severe purulent lesions (53). Since the NMR immune system at different age was not yet systematically studied, it is difficult to interpret its features to either neonate-like or senior-like type on the basis of bone marrow cellular composition or immune marker distribution. More studies are required to address this interesting question.

The predominance of myeloid cells in NMR peripheral immune organs (1, 2) combined with high life expectancy and the apparent absence of aging hallmarks allowed us to hypothesize that *H. glaber* developed species-specific mechanisms of myeloid cell control which limits the deleterious effects of chronic inflammation associated with aging. *In vitro* model of bone marrow-derived NMR macrophages could help uncover these underlying mechanisms.

Temperature can affect the metabolic rate and molecular dynamics of cells, therefore, different temperature requirements for cell cultures of NMR and mice complicate the comparison between the two species. To avoid this problem one previous study compared the results of metabolic tests for both NMR and mouse fibroblasts at 37°C (54). Our experiments showed that NMR bone marrow cultures had impaired differentiation at 37°C, but not at 32°C (**Figures 1B, D**). We prioritized the physiological relevance of temperature conditions in the context of the immune response and conducted polarization experiments, including a metabolic stress test, under different temperature conditions for the two species (32°C for NMR and 37°C for murine BMDM). Since poor thermoregulation is an important feature of NMR ecophysiology (27), further studies to address temperature-determined effects in the NMR immune system are required.

Our results indicate that NMR macrophages acquired the expected pro-inflammatory phenotype and transcription profiles upon activation with LPS and recombinant mouse IFNγ (**Figures 2**, **3**, **6**), while RNS production via inducible NO-synthase was limited (**Figures 3**, **4**). This, in turn, may be related to impaired mitochondrion fission (**Figure 3A**) and to more sustainable OXPHOS (**Figures 2C–I**) observed in polarized NMR M1 macrophages. Aerobic glycolysis is the hallmark of activated immune cells. Interestingly, recent studies of NMR metabolism under hypoxia led to the hypothesis that in most NMR tissues glycolysis and OXPHOS are strongly coupled to avoid either systemic or local lactic acidosis (32). In agreement with this idea NMR M1 cells lacked acidosis (**Figure 2I**). On the other hand, the above hypothesis did not take into consideration a possible role of NO and its derivatives in regulation of aerobic glycolysis and OXPHOS (21). Peroxynitrite is able to decrease the activity of iNOS, which could be considered as a negative-feedback regulation for RNS formation during inflammation (55). Contrary to iNOS, at the level of the mitochondrion peroxynitrite formation occurs as positive-feedback. Whereas peroxynitrite is able to exert direct influence on different elements of the mitochondrial electron-transport system, its most verse effect is to inhibit the activity of mitochondrial antioxidant enzymes with subsequent more peroxynitrite generation because of excess of ROS. Interestingly, in this study we observed reduced formation of 3-nitrotyrosine for NMR macrophages as compared to mice (**Figure 3D**), probably, due to the previously uncovered regulation mechanism of ROS production by mild depolarization of NMR mitochondria (31).

*Gch1*, encoding GTP cyclohydrolase 1, is involved in iNOS co-factor tetrahydrobiopterin (BH4) formation. BMDM obtained from *Gch1* and *Nos2* knockout mice showed that basal and ATP-linked respiration were largely maintained in M1-polarized cells in the absence of NO, moreover, these cells had lower levels of extracellular lactate (56). NO-deficient murine BMDM actively produced itaconate and IL-1β. This was further supported by transcriptomic analysis of NMR BMDM, which identified *Acod1* and *Il1b* among top-10 upregulated genes in M1 vs M0 (**Figure 6D**, **Supplementary Table 1**). However, DEG analysis of mouse datasets (GSE103958 (47)) revealed similar logFC values for these genes in M1 compared to M0 (**Supplementary Table 3**). Changes in the expression of arginine metabolism-associated genes were altered in NMR BMDM as compared to murine macrophages. NMR genome carries a functional *Nos2* gene, as its ortholog was annotated in the last version of NMR genome. We were able to detect reads differentially aligned to *Nos2*, as well as *Nos1* and *Nos3* in non-macrophage RNA-seq datasets for NMR, however, we failed to detect any expression or functional activity of this gene in BMDM. Furthermore, no changes occurred in *Slc7a2* expression in M1 (**Figure 6**, **Supplementary Table 1**), the gene encoding arginine transporter CAT2. *Slc7a2* expression increase is strongly associated with LPS/IFNγ response in mice and is involved in regulation of NO production by macrophages (47, 57, 58). *Gch1* was not upregulated in NMR M1 macrophages in contrast to M1-polarized murine BMDM (**Figure 6**, **Supplementary Table 1**, **3**). Reverse modulation of certain urea cycle and aspartate-argininosuccinate shunt genes (*Ass1*, *Asl*, *Slc25a15*) in NMR macrophages compared to mouse BMDM was noted (**Figure 6G**). Thus, the limited NO production in NMR cells under stress could be substantiated by transcriptional or post-transcriptional regulation and modulation of metabolic pathways. RNS control was reported for NMR neurons undergoing reoxygenation stress (59). We propose that similar mechanisms exist for RNS produced in response to inflammatory stimuli.

Previous *in vivo* studies with LPS-toxicity revealed atypical behavioral response to LPS-induced inflammation in NMR. Unlike in mice (60), LPS challenge did not repress locomotion or cause anxiety. At the same time, weight loss and diminished social interactions were reported, suggesting that LPS may alter certain behavioral patterns (61). Transcriptional profiling of blood and spleen cells after 4 hours following LPS challenge established similar gene expression changes in mice and NMR, including the canonical NF-κB pathway activation (1). Despite the described observations in behavioral and transcriptomic changes in NMR following LPS administration, NO production was not previously evaluated under systemic LPS-induced inflammatory conditions. Here we tested a dose of LPS (50 mcg/mice), which caused a reproducible increase in blood monocyte NO production in mice. In agreement with *in vitro* data, we did not detect NO production in NMR blood monocytes at 24 h following LPS administration (**Figure 4**). This lack of NO production in NMR monocytes *in vivo* supports functional importance of NO reduction in macrophages observed *in vitro*. This type of NMR adaptation to systemic inflammatory stimulus is probably needed for the control of NO-dependent downstream molecular pathways. Interestingly, a recent comparative study of endotoxin response in deer mouse reported a similar low *Nos2* expression in the blood, highlighting species-specific involvement of this pathway in systemic inflammation (62). A comparative analysis of transcriptomes from LPS-activated bone marrow macrophages for seven species (rat, two monogastric and four ruminant species) also demonstrated a divergent expression of arginine metabolism-associated genes (63). The authors suggested that this was due to the divergent evolution of *Nos2* distal regulatory elements (63). We found at least one conserved NF-κB binding site in the promoter of the NMR *Nos2* gene, therefore the lack of NO induction upon LPS activation may be determined by other regulatory regions or by entirely different molecular mechanisms, which require further research.

Previously published work on NMR macrophage polarization discussed the predisposition of NMR macrophages to anti-inflammatory properties. A small change in the expression of M2-associated markers in response to murine or human IL-4 could indicate that M0 macrophages population was initially closer to the M2 phenotype (35). However, in that study the authors used peritoneal macrophages, which are more M2-prone than BMDM (64). Transcriptomic analysis of BMDM showed similarity between M0 and M2 NMR macrophages with regard to their gene expression profiles (**Figures 6B, C**) and the lack of clear M2-signature (**Supplementary Table 4**). In addition, IL-4-activated NMR macrophages did not resemble mouse M2 cells in terms of their metabolic profile (**Figure 5**, **Supplementary Figure 2A**). On the other hand, chemokine expression by NMR macrophages in response to IL-4 was significant, both at transcription and protein levels (**Figures 6A, E**), arguing for at least partial activation of these cells. Pairwise alignments of protein sequences showed similarity between murine and NMR IL-4 as ~50-60% (depending on the selected *H.glaber* genome annotation). Human recombinant IL-4 also contains ~50% similar amino acids as compared to NMR counterpart. IL-4Rα showed ~54%, IL-13Rα and common γ-chain – near 80% of protein sequences similarity between species. Thus, the level of differences in amino acid composition between available recombinant IL-4 and native cytokine may present a limitation of this and similar studies and should be clarified when recombinant NMR IL-4 becomes available. However, the alternative explanation for the observed impaired M2 polarization implicated species-specific adaptations in the NMR immune system.

We also used cAMP as a non-specific M2-phenotype activator (65) to distinguish whether the overall anti-inflammatory signature or exclusively IL-4-downstream signaling is altered in NMR. *Arg1* levels were not significantly increased upon cAMP activation in NMR macrophages, while primary murine BMDM clearly upregulated *Arg1*, according to RT-PCR data (**Supplementary Figure 4**). Furthermore, cAMP did not induce visible fusion and mitochondrion mass increase in NMR cells (**Figure 5I**). Thus, we conclude that under our experimental conditions (temperature of 32°C, and DMEM/FBS medium) NMR macrophages poorly differentiated towards conventional M2 phenotype. Interestingly, NMR genome bears some peculiar features that may be related to the formation of М2 phenotype. First, *Arg1* gene contains *Caviomorpha*-specific substitution of histidine in position 254, which can potentially affect the activity of the enzyme (66). Together with NO-metabolism peculiarities discussed above (**Figure 6**) this foreshadows a possible adaptive NMR-specific reorganization of the NO-urea cycle. Another M2-characteristic Retnl cluster, which encodes resistin-like proteins in mice (*Retnla* or *Fizz1*), and is involved in regulation of alternatively-activated mouse macrophages (67), is missing in NMR genome (68).

Taken together, our study for the first time addresses immunometabolic changes in NMR macrophages upon polarization using the well-characterized *in vitro* model of BMDM with the focus on species-specific differences. The naked mole rat represents a mammal adapted to harsh ambient conditions such as low O_2_ and high CO_2_ levels in their nests, no access to water and need to dig through hard soil. Therefore, NMR tissues, including the immune system, inherit the traces of these adaptations: for instance, tight ROS and RNS production control as well as their reciprocal regulation can be linked with the need of tissue damage control and regeneration under reoxygenation. On the other hand, little is known about infections that accompanied the evolution of the naked mole rat. Presumably, a low basal metabolism and reduced thermoregulation, as well as a set of specific pathogenic or opportunistic microbiota led to selection of host tolerance over resistance mechanisms in NMR, including downregulation of inflammation-dependent RNS. Thus, long-lived, cancer-resistant and hypoxia-tolerant naked mole-rat is a promising model to study immune system evolutionary adaptations and their influence on immunosenescence.

## Data availability statement

The datasets presented in this study can be found in online repositories. The names of the repository/repositories and accession number(s) can be found in the article/**Supplementary Material**. This data can be found here: https://www.ncbi.nlm.nih.gov/bioproject/PRJNA933639.

## Ethics statement

The animal study was reviewed and approved by Local ethics committee of the “Landesamt für Gesundheit und Soziales”, Berlin, Germany (#ZH 156 and #T 0073/15) and Bioethics Committee of the EIMB RAS (Protocol No. 3 from 27/10/22).

## Author contributions

EAG, EOG, TY, MM and MV performed experiments. MV, TH, SH, OA provided critical materials. ED, AD performed RNA-sequencing and bioinformatic analysis. EAG, EOG, MD, MV and SN designed experiments, discussed results and wrote the manuscript. All authors contributed to the article and approved the submitted version.
