# Supplementary material for: Macrophages from naked mole-rat possess distinct immunometabolic signatures upon polarization
Source: Front Immunol. 2023 Apr 19;14:1172467. doi: 10.3389/fimmu.2023.1172467 (PMC10154529; doi:10.3389/fimmu.2023.1172467)
Supplement: Supplementary file 1 [file DataSheet_1.pdf]

Supplementary Material

Macrophages from naked mole-rat possess distinct immunometabolic signatures upon polarization

E.A. Gorshkova*, E.O. Gubernatorova, E.M. Dvorianinova, T. R. Yurakova, M.V. Marey, O.A. Averina, S. Holtze, T. Hildebrandt, A.A. Dmitriev, M.S. Drutskaya, M.Yu. Vyssokikh, S.A. Nedospasov*

*** Correspondence:**

E.A. Gorshkova: gorhsama@gmail.com

S.A. Nedospasov: sergei.nedospasov@gmail.com

1.1 Supplementary Tables

Supplementary Tables 1-4 uploaded as data sheets (Excel files).

Supplementary Table 1 – Hgl M0 vs M1 Differentially Expressed Genes

Supplementary Table 2 - Hgl M0 vs M2 Differentially Expressed Genes

Supplementary Table 3 - Mmu M0 vs M1 Differentially Expressed Genes

Supplementary Table 4 – Mmu M0 vs M2 Differentially Expressed Genes

Supplementary Table 5 – Gene Set Enrichment analysis of DEGs

1.2 Supplementary Figures

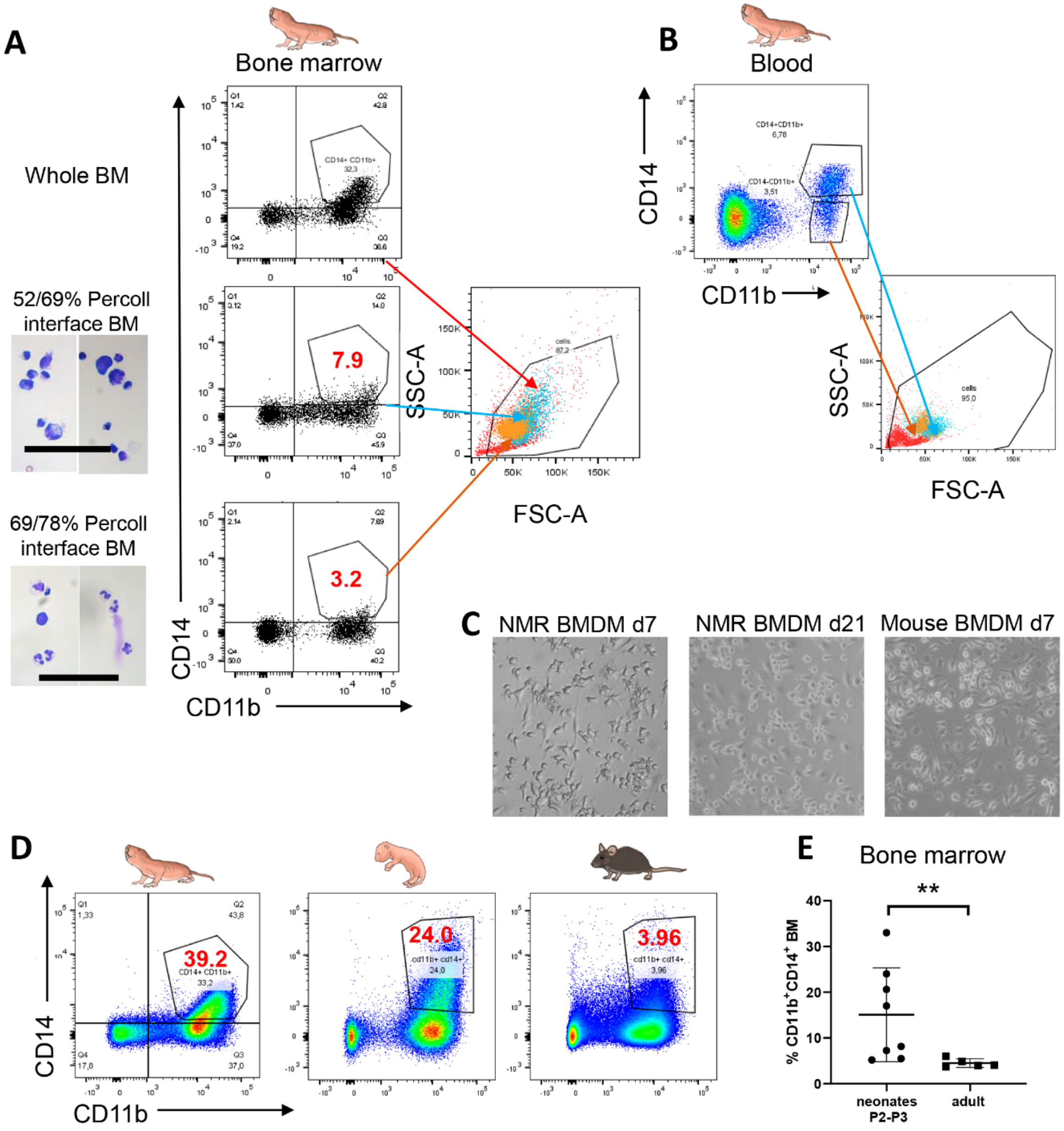

Supplementary Figure 1. Expression of CD11b and CD14 by myeloid cells in NMR and mouse bone marrow and blood.

(**A**) NMR bone marrow cells were fractioned by gradient centrifugation (52/69/78% percoll gradient) and analyzed by FACS and light microscopy. The majority of $CD11b^+$ $CD14^+$ cells were present in 52/69 fraction (blue arrow), which was enriched with myeloid progenitors and monocytes; 69/78 fraction was enriched with mature neutrophils (orange arrow). Smears of BM fractions were stained by May Grünwald-Giemsa method, scale bar 100 µm. (**B**) $CD11b^+$ $CD14^+$ population in NMR blood apparently represents monocytes (blue arrow). (**C**) Microscopic photo of 7 and 21 day old NMR BMDM cultures and 7 day mouse BMDM culture. (**D**) Representative dot plots of NMR, neonate and adult mouse bone marrow stained with a-CD14, a-CD11b Abs. (**E**) Percent of $CD11b^+$ $CD14^+$ cells in bone marrow of neonate mouse were increased as compared to adult mice. Results are shown as mean ± SD; **$p<0.01$, Student's unpaired t-test.

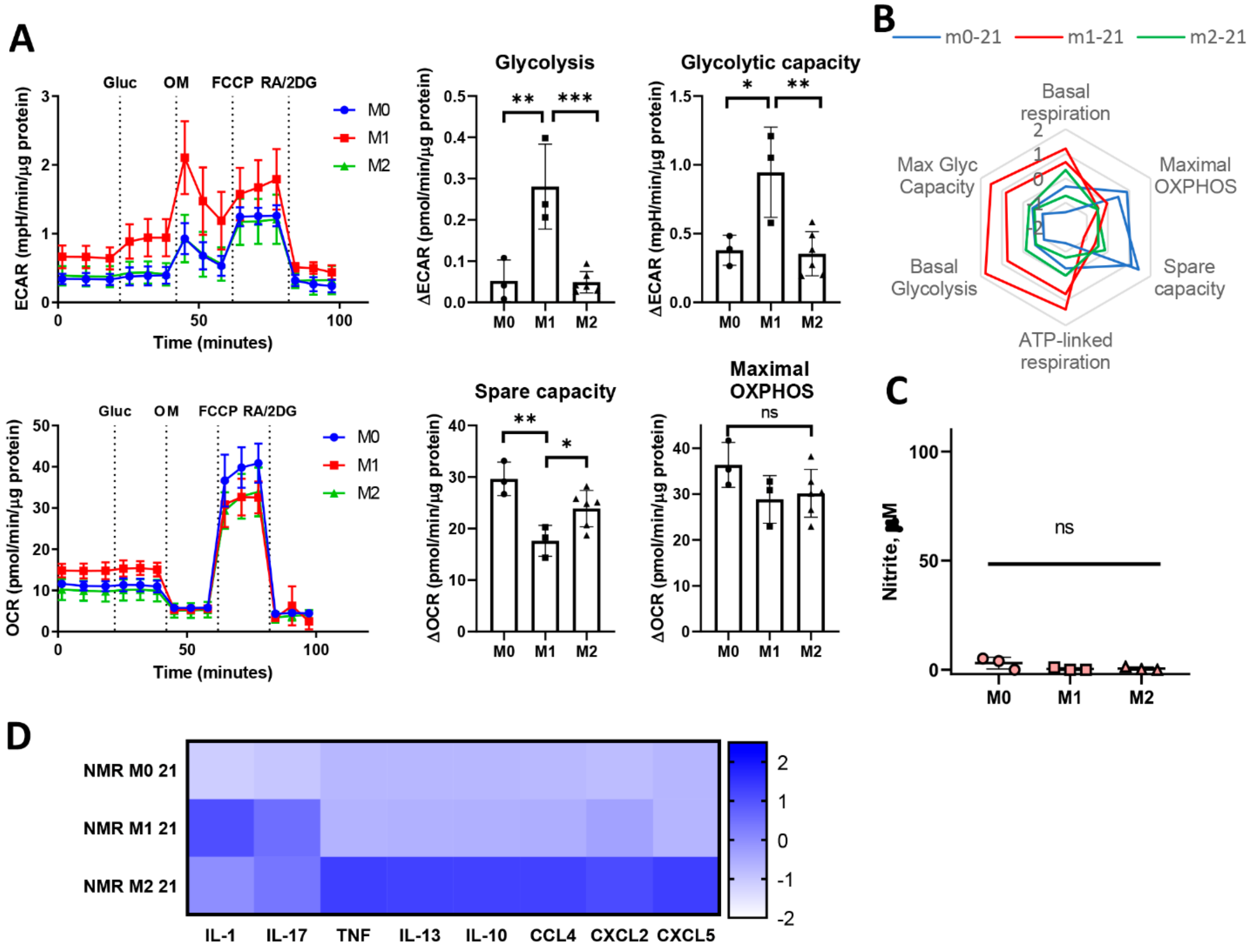

Supplementary Figure 2. 21-day cultured NMR BMDM share similar phenotypes with 7-day cultured NMR BMDM upon polarization.

(**A**) Representative diagrams of the real-time changes in OCR and ECAR of M0 (blue), M1 (red) and M2 (green) NMR macrophages under combined stress test. (**B**) Summary of metabolic profiles of 21-day cultured NMR M0 (blue), M1 (red) and M2 (green) macrophages presented as z-score transformed mean for six metabolic parameters on radar chart, each line on a diagram represents an independent metabolic stress experiment. (**C**) Nitrite concentration in supernatants of 21-day NMR BMDM activated by LPS (10 ng/mL) and mouse recombinant IFNγ (10 ng/mL) (M1) or mouse recombinant IL-4 (M2) for 24 h, results of a representative experiment. (**D**) Multiplex immunoassay on the samples of M0, M1 and M2 supernatants of NMR (n=4), each cell shows mean of z-score transformed cytokine concentration in medium (pg/mL). Results displayed as mean ± SD; ns – non-significant; ****p<0.001, one-way ANOVA test.

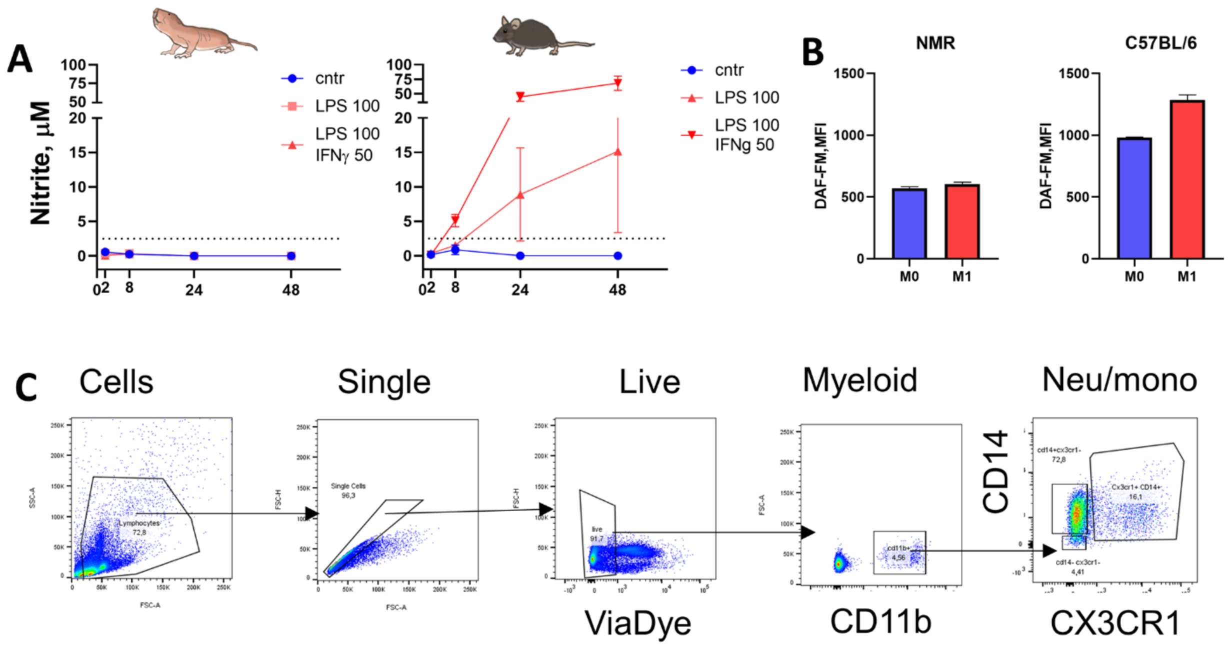

Supplementary Figure 3. NO production by NMR macrophages and blood monocytes.
(**A**) Nitrite concentrations in medium collected after 2, 8, 24, and 48h of activation with LPS (100 ng/mL) or LPS (100 ng/mL)/IFNγ (50 ng/mL) cocktail in NMR (left) and mouse samples (right).
(**B**) DAF-FM MFI in NMR and mouse M0 and M1 BMDM activated for 24 h with LPS/IFNγ.
(**C**) Gating strategy for FACS analysis of blood samples following LPS administration.

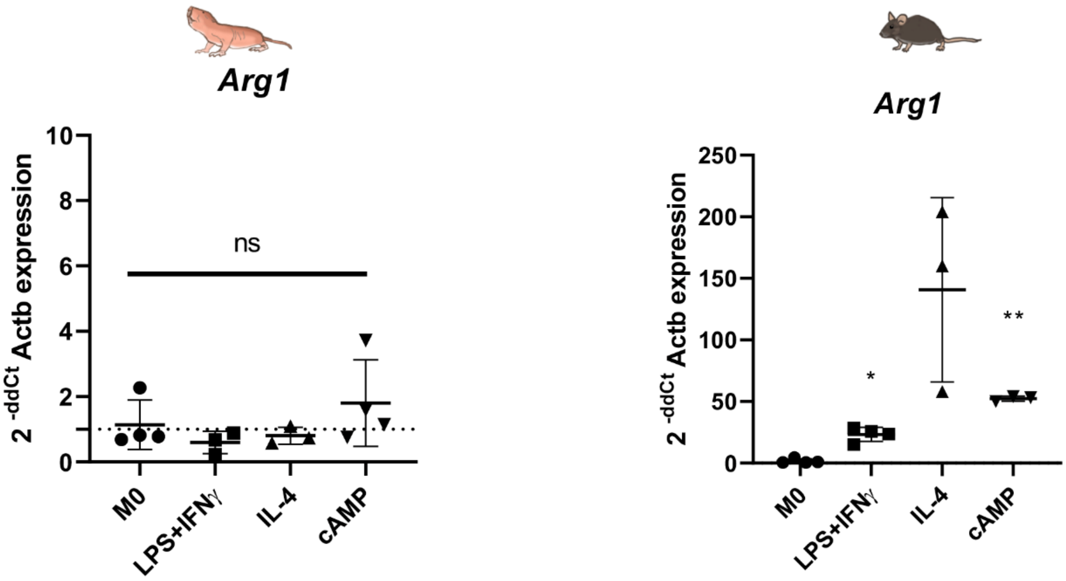

Supplementary Figure 4. *Arg1* expression in NMR BMDM is not upregulated in response to cAMP

Relative expression of *Arg1* in M1 and M2-conditioned NMR and murine BMDM as a fold change to the expression level observed in M0 ($2^{-\Delta\Delta Ct}$). Results displayed as mean ± SD; ns – non-significant; ****$p<0.001$, mixed model ANOVA test.

.

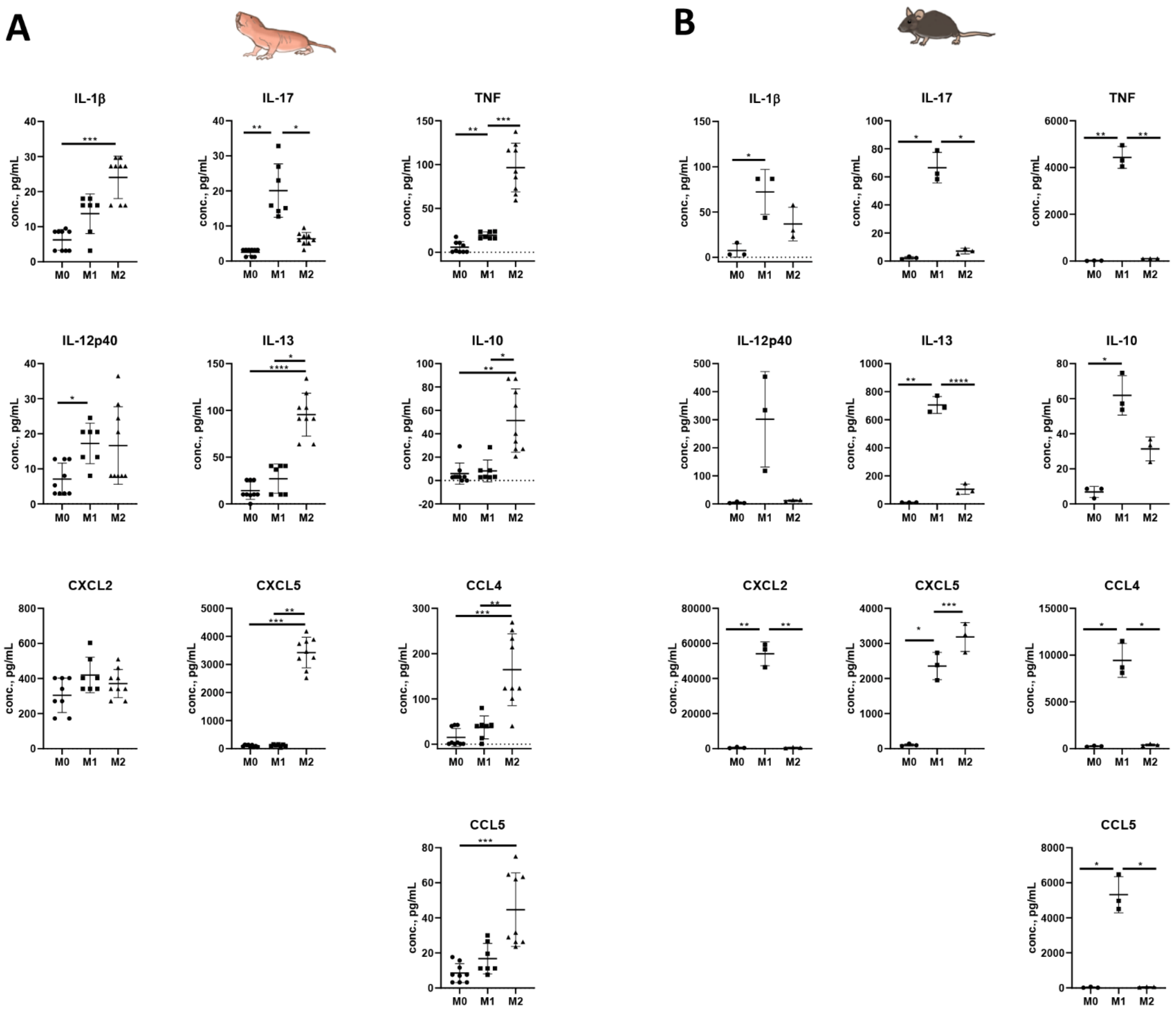

Supplementary Figure 5. Secretion of cytokines and chemokines by NMR and murine M0, M1, M2 macrophages.

Multiplex immunoassay on the samples of M0, M1 and M2 supernatants of NMR (A) and (B) mouse macrophages. Results displayed as mean ± SD; *p<0.05;**p<0.01;***p<0.001;****p<0.0001; one-way RM ANOVA test.
